# Editorial: Theranostic nanomaterials

**DOI:** 10.3389/fbioe.2023.1273060

**Published:** 2023-08-14

**Authors:** Wansong Chen, Yue Zhang, Jianhua Zhang, Long Wang, Chengcheng Niu

**Affiliations:** ^1^ Department of Ultrasound Diagnosis, The Second Xiangya Hospital, Central South University, Changsha, China; ^2^ Department of Orthopedics, Xiangya Hospital, Central South University, Changsha, China; ^3^ Hunan Provincial Key Laboratory of Micro and Nano Materials Interface Science, College of Chemistry and Chemical Engineering, Central South University, Changsha, China; ^4^ School of Engineering, Westlake University, Hangzhou, China; ^5^ Department of Polymer Science and Engineering, School of Chemical Engineering and Technology, Tianjin University, Tianjin, China

**Keywords:** theranostics, nanomaterials, bioimaging, therapy, drug delivery

The effective treatment of diseases requires the combination of diagnosis and therapy, which has become one of the important directions in the field of biomedicine. Theranostic nanomaterials can achieve this goal by loading both drug molecules and diagnostic reagents. Theranostic nanomaterials can also improve the targeting and precision of diagnosis and therapy through surface modifications. The commonly used theranostic nanomaterials include liposomes, polymer nanoparticles, inorganic nanoparticles and organic/inorganic hybrid nanomaterials. Various diagnostic methods have been developed with theranostic nanomaterials, such as ultrasound imaging, radiography, fluorescence imaging and photoacoustic imaging. However, the clinical applications of theranostic nanomaterials faces some challenges due to the complexity of the physiological environment *in vivo* and the diversity of diseases. Firstly, the imaging signals of theranostic nanomaterials are lack of sufficient specificity, penetrating depth, and resolution. As a result, it is challenge to detect small lesions at the early stage of diseases. It is necessary to optimize the specificity and sensitivity of disease diagnosis, especially for occult and early lesions. Secondly, the therapeutic activity of theranostic nanomaterials is expected to be adjusted according to the diagnosis results, so as to achieve personalized treatment. However, the majority of the current theranostic nanomaterials lack flexible means to adjust the therapeutic activity post their administration. Thirdly, traditional small molecule drugs are prone to cause drug resistance in target tissues. Nanomedicines with high efficacy and low risk of drug resistance are highly desirable for the disease treatments. In addition, the exact mechanism of theranostic nanomaterials in body is still poorly understood. Although possible mechanisms can be inferred through a series of *in vitro* studies, the biological environment *in vivo* is much more complex than that *in vitro*. More endevours are required to disclose the acting mechanism of theranostic nanomaterials, which will be helpful for their future design and optimization.

Based on the above challenges, this Research Topic covers the latest research progress in theranostic nanomedicine. In terms of diagnostics, Kakaei et al. reviewed the recent progress and challenges in perfluorocarbon nanomaterials for theranostic applications. The oxygen dissolving ability of perfluorocarbons allows them to act as oxygen carriers and enable ultrasound imaging. Moreover, with incorporation of magnetic resonance contrast agents and photothermal agents, perfluorocarbons can also achieve magnetic resonance imaging and photoacoustic imaging *in vivo*. The oxygen delivered by perfluorocarbons can alleviate the hypoxia in the lesions of diseases such as tumors, thrombosis, arthritis, and muscle atrophy.

In terms of disease therapy with theranostics nanomaterials, Nie et al. reported the use of selenium-based agents for radiosensitization of lung cancer. Selenium compounds and nanoselenium induced lung cancer cell apoptosis by blocking G2/M cell cycle arrest, thus enhancing the efficacy of radiotherapy. Wu et al. reported perfluorane-loaded liposomal microbubbles for antitumor immunotherapy under low intensity pulsed ultrasound. The cavitation effect induced by ultrasound microbubbles disrupted tumor blood vessels and facilitated the infiltration of immune cells into the tumor, triggering a sustained antitumor immune response. Besides tumor therapy, theranostic nanomedicines have also been applied in other diseases. Zhang et al. prepared poly (lactic-co-glycolic acid) (PLGA) nanoparticles loaded with sulfasalazine to inhibit neointimal hyperplasia by inducing autophagy. The inhibition of neointimal hyperplasia by sulfasalazine loading PLGA nanoparticles was through NF-kB/mTOR-mediated autophagy. Yan et al. used PLGA nanoparticles as carriers to deliver growth factors and controlled their release process by low-intensity pulsed ultrasound. Due to the controlled release of growth factors and physical stimulation by ultrasound, PLGA nanoparticles loaded with growth factors significantly promoted periodontal bone regeneration.

In summary, theranostic nanomaterials can combine imaging agents and therapeutic drugs into one nanoplatform to achieve disease theranostics. Moreover, the therapeutic process of theranostic nanomedicines can be tunned with the assistance of external stimulus such as ultrasound. The bioimaging functions of theranostic nanomaterials are helpful to understand their therapeutic activity in biological environment. All these two aspects endow theranostic nanomaterials with great potential in the treatment of various diseases, such as cancer, cardiovascular diseases and bone diseases. Many challenges remain to be overcome prior to their clinical translation, such as biocompatibility, biodistribution, bioavailability and biosafety. Therefore, more efforts are needed to develop novel nanomaterials and strategies for theranostics in the future. We hope that this project will stimulate the interest of researchers in chemistry, materials, biomedicine and other disciplines, aiming to promote the development of theranostic nanomaterials.

